# Data Correction of Intensity Modulated Small Angle Scattering

**DOI:** 10.1038/s41598-019-44493-9

**Published:** 2019-06-12

**Authors:** Fankang Li, Steven R. Parnell, Robert Dalgliesh, Adam Washington, Jeroen Plomp, Roger Pynn

**Affiliations:** 1 Neutron Sciences Directorate, Oak Ridge National Laboratory, Oak Ridge, TN 37830 USA; 2 Faculty of Applied Sciences, Delft University of Technology, Mekelweg 15, Delft, JB 2629 The Netherlands; 3 ISIS Pulsed Neutron and Muon Source, STFC, Rutherford Appleton Laboratory, Chilton, Oxon, OX11 0QX UK; 4 Center for Exploration of Energy and Matter, Indiana University, Bloomington, IN 47408 USA

**Keywords:** Characterization and analytical techniques, Characterization and analytical techniques, Spectrophotometry

## Abstract

To investigate long length scale structures using neutron scattering, real space techniques have shown certain advantages over the conventional methods working in reciprocal space. As one of the real space measurement techniques, spin echo modulated small angle neutron scattering (SEMSANS) has attracted attention, due to its relaxed constraints on sample environment and the possibility to combine SEMSANS and a conventional small angle neutron scattering instrument. In this report, we present the first implementation of SEMSANS at a pulsed neutron source and discuss important corrections to the data due to the sample absorption. These corrections allow measurements made with different neutron wavelengths and SEMSANS configurations to be overlaid and give confidence that the measurements provide an accurate representation of the density correlations in the sample.

## Introduction

Small angle scattering (SAS), using either X-ray or neutrons, has made important measurements of the structures of many materials including polymers^1^, magnetic materials^2^ and biological materials^3^, addressing length scales from 1 nm to several hundreds of nm. SAS works in reciprocal space and measures the deflection of collimated radiation away from its initial trajectory^4–8^. Hence it becomes more and more challenging as the scattering angle decreases. For neutrons, the neutron spin provides us with another option to label the neutron’s trajectory change with high precision. In a given magnetic field, the neutron spin will execute a motion called Larmor precession^9^, and the accumulated Larmor phase is given by

$${\rm{\Phi }}=\frac{\gamma m}{h}\lambda {\int }_{path}\,Bdl\propto FI\cdot \lambda ,$$

where *γ* is the gyromagnetic ratio of the neutron, *m* is its mass, *h* is the Planck constant, *λ* is the neutron wavelength and *FI* is the field integral along the neutron trajectory. Using a neutron spin analyzer, the projection of the polarization vector that is parallel to the analyzing direction can be selected and analyzed, yielding the cosine of the Larmor phase $P=\,\cos \,{\rm{\Phi }}$. Any technique that can correlate the change in the neutron energy or momentum with Φ is called neutron Larmor labeling.

The first technique of this type was introduced by F. Mezei in 1972^10^, called neutron spin echo (NSE). It is typically used to label the neutron energy change in quasi-elastic scattering with high resolution. To label the neutron momentum or trajectory change in elastic neutron scattering, two methods have been implemented, spin echo small angle neutron scattering (SESANS)^11^ and spin echo modulated small angle neutron scattering (SEMSANS)^12–15^. Though the similarities and difference between these two techniques have been discussed before^14^, some important details about data analysis, especially for time-of-flight (TOF) measurements, have not been discussed.

**Figure 1:**
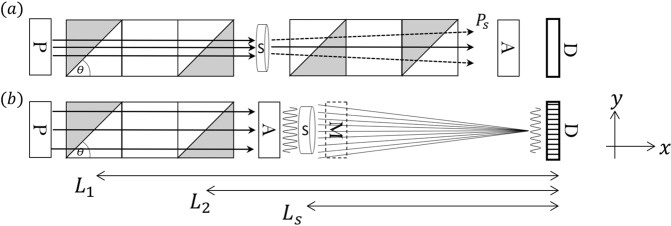
(**a**) The SESANS setup, where different shaded areas correspond to different magnetic field directions. The scattered neutrons are indicated by the dashed lines. (**b**) The SEMSANS setup. The second WP has higher and opposite field compared with the first one. After the sample, only the trajectories of the scattered neutrons captured by a specific pixel of the detector are plotted for clarity. Boxes labeled with *A*, *P* and *D* indicate the neutron polarization analyzer, polarizer and detector. *M* denotes the neutron monitor right behind the sample used for the transmission measurement when needed.

The magnetic field boundary of NSE is perpendicular to the beam direction, which means the Larmor phase is not sensitive to any trajectory change to the first oder. The principle of SESANS is to introduce a tilted magnetic field boundary with different field directions on the two sides of the field boundary to introduce the sensitivity to the neutron trajectory change^16^. There are several ways to implement the tilted field boundary, including radio frequency (RF) flippers^17^, permalloy films^18^ and magnetic Wollaston prisms (WP)^19–22^. Taking the WP as an example, the SESANS setup is shown in Fig. 1(a). The magnetic field configurations after the sample are flipped compared with the fields before the sample. With this setup, it is possible to measure the difference of the Larmor phase accumulated before and after the sample. When there is no scattering, no difference in phase will be measured thus no polarization change will be observed on the detector. But when scattering occurs within the sample, the trajectory after the sample will deviate from its initial path such that the field integral cannot be balanced any more and hence a net polarization change will be measured.

1 $${P}_{n}(\xi )=\frac{{P}_{s}(\xi )}{{P}_{b}(\xi )}={e}^{\sigma t(G(\xi )-1)}$$

2 $$G(\xi )=\frac{1}{\sigma {k}_{0}^{2}}{\int }_{{Q}_{\min }}^{{Q}_{\max }}\frac{d\sigma }{d{\rm{\Omega }}}(Q)\cos \,({Q}_{y}\xi )d{Q}_{y}d{Q}_{z}$$

3 $$\sigma =\frac{1}{{k}_{0}^{2}}{\int }_{{Q}_{\min }}^{{Q}_{\max }}\,\frac{d\sigma }{d{\rm{\Omega }}}(Q)d{Q}_{y}d{Q}_{z}$$

The measured polarization of SESANS is well-known as equation (1), where *P*_*b*_, *P*_*s*_ are the polarization measured with the blank to remove the instrumental errors and sample respectively. *ξ* is termed spin echo length, which is the length scale accessed in real space. *t* and *k*_0_ denote the sample thickness and incoming neutron wave vector respectively. *G*(*ξ*) is the correlation function of the sample, as given in equation (2), which basically is the cosine Fourier transform of the differential scattering cross section $\frac{d\sigma }{d{\rm{\Omega }}}(Q)$ with Q being the momentum transfer of neutrons. *Q*_*min*,*max*_ provides the *Q* range where the Fourier transform is performed for a real instrument. *σ* denotes the single neutron scattering cross section, given in equation (3). So SESANS is a 1-D real space technique and it provides a one to one relation between the density correlation function of the sample and the neutron polarization observed after Larmor labeling. Clearly, any neutron absorbed by the sample would not contribute to the measured polarization, or to the value of *G*(*ξ*). SESANS is only sensitive to the direction parallel to the gradient of the magnetic field integral (*y* direction in Fig. 1). As shown by Rekveldt^23^, both SESANS and SEMSANS will directly yield the pair correlation function even for strongly scattering samples with significant multiple scattering. This makes them extremely useful for systems with strong scattering to measure inter-particle correlations. This is different from that of SANS, which is typically used for dilute systems for form factor measurements.

## Principle of Spin Echo Modulated Small Angle Neutron Scattering

For SEMSANS, as shown in Fig. 1(b), half the number of Wollaston prisms required for SESANS are used, with the second WP generating a higher field (*B*_2_) than the first one (*B*_1_). Due to the field difference between the two WPs, the Larmor phase (Φ) varies across the transverse direction (*y*) of the neutron beam, which will introduce a modulation of polarization vectors as $P(y)=\,\cos \,{\rm{\Phi }}(y)$. After going through the polarization analyzer, an intensity modulation will be observed on the detector. Due to the divergence of the neutron beam, the modulation might have low visibility. However, by satisfying *B*_1_*L*_1_ = *B*_2_*L*_2_ with *L*_1_ and *L*_2_ being the distances of the WPs to the detector, the induced Larmor phase dispersion on every single point of the detector can be minimized such that a high visibility intensity modulation can be obtained^14^.

As shown in Fig. 1(b), due to the scattering of the sample, the neutron trajectories will be deviated from their initial paths such that the visibility of the modulation will be lowered because each pixel on the detector will capture neutrons scattered from different points on the sample^14^. When tracing back from a specific pixel of the detector, neutrons with different momentum transfers will be detected and thus Fourier transformed by the modulation, as shown in Fig. 1(b). The trajectory change is encoded and reflected as a change in the amplitude of the intensity modulation measured by the detector, instead of as a polarization change in SESANS. The ratio of the modulation amplitudes for a sample and a blank with the same absorption, *A*_*s*_ and *A*_*b*_ respectively, is given by equation (4). The accessible spin echo length in this case is given by $\xi =\frac{\lambda \omega {L}_{s}}{2\pi }$ with *ω* being the frequency of the spatial modulation and *L*_*s*_ being the distance from the sample to the detector, as given in Fig. 1(b). For SEMSANS, polarization is just a way to produce the intensity modulation at the detector. Therefore, any technique that can produce an intensity modulation can be used for similar purposes. For example, a Talbot-Lau interferometer with gratings has also been demonstrated to measure the correlation function of a sample^13^.

4 $${A}_{n}(\xi )=\frac{{A}_{s}}{{A}_{b}}={e}^{\sigma t(G(\xi )-1)}$$

Unlike SESANS, where sample absorption does not affect the neutron polarization, the absorption of the sample will cause damping of the intensity modulation of SEMSANS. This is especially troublesome for TOF SEMSANS, where the wavelength dependence of the absorption efficiency will contribute to the correlation function measured, if not considered properly. Though preliminary data of TOF SEMSANS has been reported in ref.^12^, the data reproducibility with different magnetic fields is missing and the discussion of the absorption correction is still vague, which makes it difficult to implement on a real instrument. Since the polarization analyzer can be placed before the sample, a method of combining SANS and SEMSANS^24^ has been proposed to use the neutrons usually wasted at the beam stop to further increase the accessible length scales. As the first step, it is critical to have a way of correcting the SEMSANS data such that the wavelength-dependent absorption efficiency can be removed properly.

For SESANS or SEMSANS, equation (1) is true only when all the scattering from the sample can be collected such that a full Fourier transform from *Q*_*min*_ = 0 to *Q*_*max*_ = ∞ in equation (2) can be obtained. In practice, this is difficult mainly due to: (1) the limited aperture of the detector or polarization analyzer and (2) the limited acceptance solid angle of the encoding devices. On the other hand, for SEMSANS, the Q range is mainly restricted by (1) the distance of the detector to the sample, (2) the size of the sample and (3) the divergence of the incident neutron beam. With an infinitely large sample or source, every single point of the detector will provide the same correlation function. However, for a real sample of finite size, part of the scattering may be missing. This will cause a spatial dependence of the Q range coverage across the detector. So it is important to keep this in mind when designing an experiment. Since the Q range coverage for SEMSANS has been qualitatively presented and discussed in ref.^25^, in this report, we will only show the correction of the SEMSANS data due to the sample absorption, assuming the Fourier transform is relatively complete, which is reasonable since the intensity of the scattering drops down quickly towards higher Q. The issue regarding the range of the Fourier transform will be discussed quantitatively in a latter article.

## Principle of Absorption Correction for SEMSANS

Following equation (4), the ideal way of conducting a SEMSANS measurement is to introduce a blank sample with the same absorption efficiency but no scattering as the sample of interest. But in reality, it is difficult and sometime impossible to find such a blank sample. So in many cases, a blank, such as air or solvent, is preferred. For a sample with different absorption efficiency from the blank, the measured amplitude of the modulation of the sample needs to be renormalized by its absorption efficiency as,

5 $${A}_{n}(\xi )=\frac{{A}_{s}}{T{A}_{b}}$$

*T* denotes the attenuation efficiency of the sample with respect to the blank. To measure *T*, the best approach is to introduce a neutron monitor right after the sample to measure all the neutrons passing through the sample and blank such that

$$T=\frac{{N}_{s}(\lambda )}{{N}_{b}(\lambda )},$$

where *N*_*s*,*b*_ are the neutron counts for sample and blank respectively. For some results we have measured, which will be discussed in the following section, no monitor was implemented. Instead, we used the same position sensitive detector used for the measurement of the intensity modulation. We sum up the two spin states and integrate the whole detector to obtain *T* as

$$T=\frac{{N}_{+,s}+{N}_{-,s}}{{N}_{+,b}+{N}_{-,b}},$$

where the superscript “+” and “−” denote the two spin states and *b*, *s* denote the blank and sample respectively. This method will give the correct result provided the active area of the detector is large enough to capture all of the attenuated direct beam and the small-angle scattering. The data corrected with these two approaches will be shown and compared in the following section.

When conducting a SEMSANS experiment, to eliminate the inhomogeneity of the beam profile, the intensity modulation of both neutron spin states (+, −) instead are measured as ${I}_{b,s}(y)=\pm \,{A}_{b,s}\,\sin (\omega y+\varphi )+{\bar{I}}_{b,s}$ and “+” is involved due to the opposite polarization between the two spin states. *A* and $\bar{I}$ are the amplitude and shim intensity of the spatial modulations respectively. By performing $P=\frac{{N}_{+}-{N}_{-}}{{N}_{+}+{N}_{-}}$, a map of the polarization modulation can be reconstructed with no dependence on the beam profile as ${P}_{b,s}=\frac{{A}_{b,s}}{{\bar{I}}_{b,s}}\,\sin (\omega y+\varphi )$. The amplitude of the polarization modulation, which is the same as the visibility of the intensity modulation, is given as ${V}_{b,s}=\frac{{A}_{b,s}}{{\bar{I}}_{b,s}}$. Using these terms, equation (5) can be rewritten as

6 $${A}_{n}=\frac{{A}_{s}}{T{A}_{b}}=\frac{1}{T}\frac{{V}_{s}{\bar{I}}_{s}}{{V}_{b}{\bar{I}}_{b}}=\frac{{V}_{s}{\rm{\Gamma }}}{{V}_{b}T}$$

**Figure 2:**
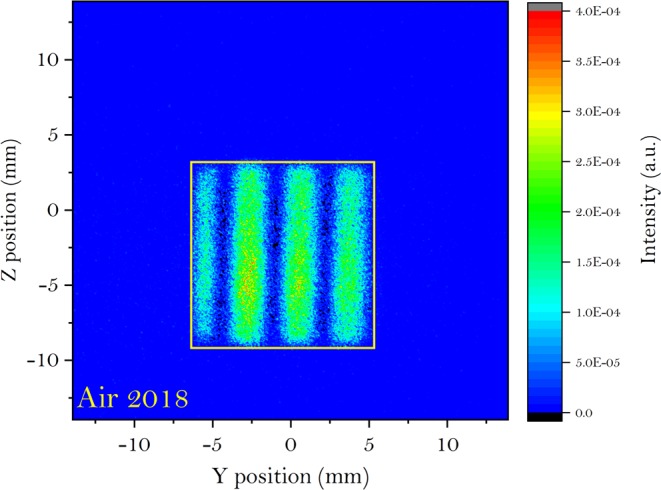
The intensity modulation of one of the spin states measured in the 2018 setup with air being the blank. The wavelength of this frame is 6.27 Å. The whole picture is all the active area of the detector and the yellow box indicates the area of interest used to calculate Γ in equation (6).

*V*_*s*_ and *V*_*b*_ can be obtained by performing a sinusoidal fit. Γ is the ratio of the shim intensity between the sample and the blank, i.e. ${\rm{\Gamma }}=\frac{{\bar{I}}_{s}}{{\bar{I}}_{b}}$, which can be obtained by summing up the two spin states over the area where the intensity modulation on the detector is analyzed. The area of interest does not have to be the area of the main beam indicated by the yellow box in Fig. 2. Instead, it could be smaller than the yellow box providing the sinusoidal fit is reasonably good. Differently, *T* is obtained by integrating the neutrons over the whole detector or by using a monitor placed directly behind the sample, as shown in 1(b).

Also, the typical way of obtaining *V*_*s*_, *V*_*b*_ is to extract the amplitude of the sinusoidal fit for a given modulation. Another option is to use standard deviation (SD) of the modulation as a quick way to evaluate the data. In the following section, the analysis with standard deviation will be performed and compared with fitting.

## Semsans Experiment at LARMOR With a Pulsed Beam

The experiments were conducted on the LARMOR beamline of the ISIS Neutron and Muon Source based at the Rutherford Appleton Laboratory in Oxfordshire, UK. Superconducting magnetic Wollaston prisms^20^, instead of the usual RF flippers installed on LARMOR, were used to generate the intensity modulations on the detector. For the following discussions, there are two experiments involved, labeled as 2016 and 2018. The samples for both experiments are 1 *mm* thick of colloidal monodisperse spherical poly(methyl methacrylate) (PMMA) nanoparticles dispersed in deuterated *d*_8_-dodecane with a volume fraction of 40%. The surfaces of the PMMA particles are coated with a (12-hydroxystearic acid) PHSA polymer brush to prevent aggregation^26^. The blank used for the normalization of both experiments is air. To measure the intensity modulation, a micro-channel plate detector^27^ was used in TOF mode. The spatial resolution of the detector is 55 *μ*m over an active area of 28 × 28 mm^27^. Two different analyzers were used during these two measurements. For 2016, a ^3^ He analyzer was used and placed after the sample to provide a uniform analyzing power for all the neutron trajectories. For 2018, a supermirror analyzer was used, which was placed before the sample to avoid the nonuniformity of the analyzing power. The collimation of the beam was defined with two pin holes separated by ~5 *m* with one upstream (20 *mm* × 20 *mm*) and one just in front of the sample (10 *mm* × 10 *mm*). For both measurements, the tuning of *B*_1_*L*_1_ = *B*_2_*L*_2_ was achieved by scanning the ratio $(r=\frac{{B}_{1}}{{B}_{2}})$ while keeping the difference (*B*_1_ − *B*_2_) the same. Benefiting from the multiple wavelengths available at LARMOR, a wide range of spin echo length can be accessed simultaneously at a single setting of magnetic fields in the WPs.

**Figure 3:**
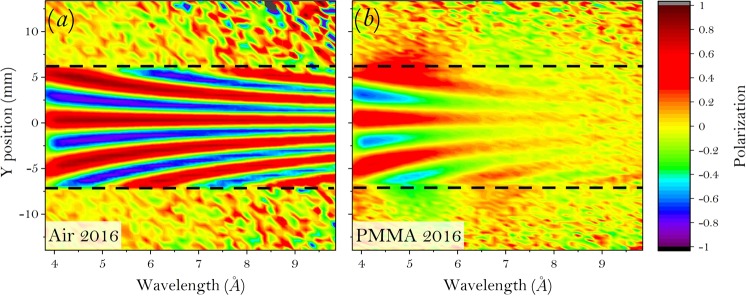
Stacked map of the 1D polarization plot as a function of wavelength, where (**a**) is the air and (**b**) is the PMMA 2016 sample. The dashed lines indicate the size of the yellow box in Fig. 2.

Figure 2 shows the intensity modulations of one of the spin states obtained in the 2018 measurements at neutron wavelength of 6.27 Å. As discussed before, both spin states are measured to eliminate any possible nonuniformity of the beam spatial profile. Then the polarization modulation is integrated vertically along *z* within the yellow box for each wavelength. The 1D polarization plots for all the wavelengths are then stacked together into a 2D map for both the air and PMMA, as given in Fig. 3. The vertical centering of the modulation in Fig. 3 can be shifted by tuning the current in the rectangular field between the WPs. While the modulation in Fig. 3(a) is sharp and clean, the scattering from the PMMA sample will smear out its visibility, as shown in Fig. 3(b). As the wavelength increases, the modulation will be smeared out more. For each wavelength, both the fit and the standard deviation (SD) are used to obtain $\frac{{V}_{s}}{{V}_{b}}$ for the data correction.

## Data Correction with the Position Sensitive Detector

**Figure 4:**
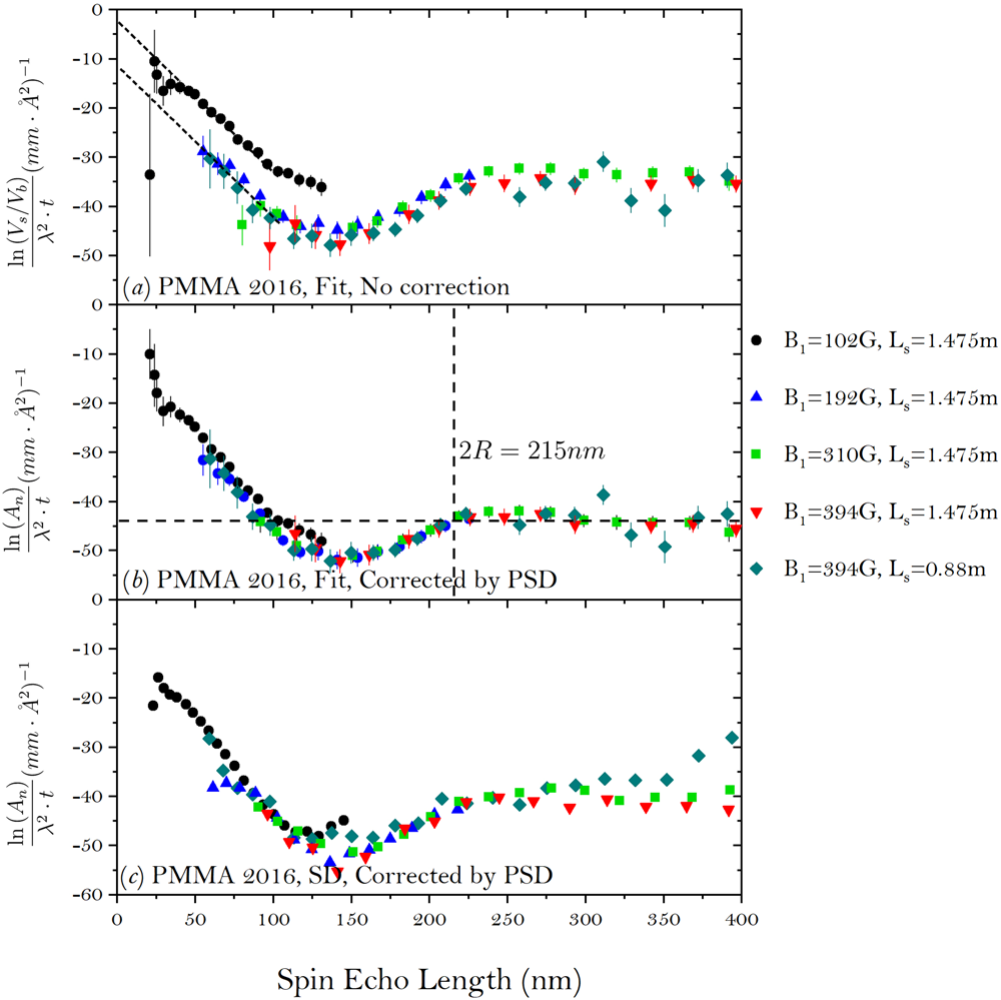
The correlation function of the colloidal PMMA sample measured with the SEMSANS setup in 2016. The setting of the magnetic field in the first WP (*B*_1_) and sample distance to the detector (*L*_*s*_) are listed in the figures. The measurement for each experimental setting took ~2 hours. (**a**) *A*_*n*_ is calculated straightly by taking ${A}_{n}=\frac{{V}_{s}}{{V}_{b}}$ and no correction is applied, (**b**) is the same as (**a**) but the data is corrected using equation (6) with the absorption efficiency measured by integrating whole detector and (**c**) the same as (**b**) but the ratio of the visibility $(\frac{{V}_{s}}{{V}_{b}})$ was obtained by using the standard deviation of the modulation.

For the 2016 experiment, the measurements were conducted at five different magnetic field settings, which are listed in the Fig. 4. The quantity $\frac{\mathrm{ln}({A}_{n})}{{\lambda }^{2}\cdot t}$ is plotted such that the wavelength dependence of the total scattering can be removed^28^. As one can see from Fig. 4(a), these curves at different settings of field do not overlap with each other, especially at the shortest spin echo length. This is due to the usage of different wavelength bands to achieve the same spin echo length with different settings of fields. During the experiment, no monitor was implemented after the sample, so the intensity on the whole detector was integrated and used to obtain *T*, as labeled by PSD in the figure. After being corrected by equation (6), the measurements with different experimental settings in Fig. 4(b) overlap with each other. The PMMA system is well understood and the average diameter of the PMMA particles can be approximately given by the first crossing point of the curve with its saturation level, which is 215 *nm*^26^. This is very close to the hydrodynamic diameter of the particles (200 *nm*) obtained from dynamic light scattering.

Also, the feasibility of using the standard deviation for the calculation of the correlation function is also evaluated. As we can see from Fig. 4(b), after correction, the measurements at the different magnetic fields and sample-to-detector distance overlap with each other well when the modulation amplitudes are obtained by fitting the data to a sinusoidal function. For the same field setting, the spread of the data points is larger compared when the fitted amplitudes are replaced by the standard deviation of the modulation. While the error for a measurement with poor statistics can be lowered by forcing the data to comply with a specific fit function, the method of standard deviation assumes no correlation between the data points and thus comes with a larger error. Also due to the poor statistics at longer wavelength, the error of the data contributes too much to the standard deviation of the data, which makes it too noisy to use standard deviation. Nevertheless, as an easy and quick way to check the data, standard deviation is still useful provided the statistics of the measurements are adequate.

## Data Correction with the Monitor

**Figure 5:**
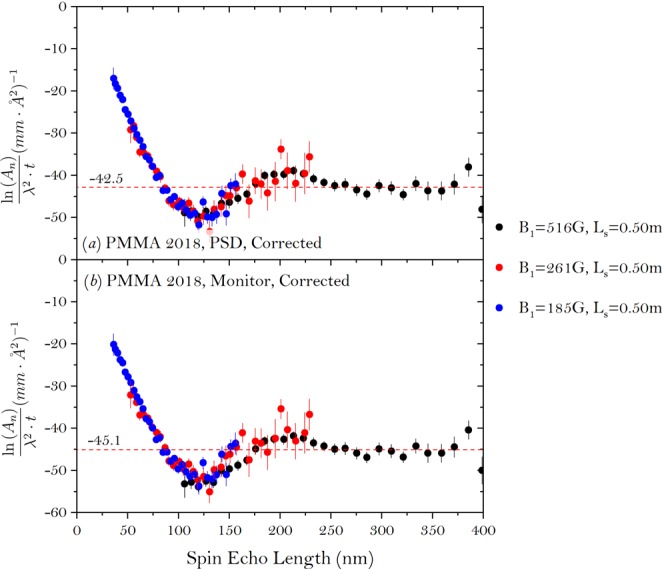
The correlation function of the colloidal PMMA sample measured with the SEMSANS setup in 2018. The setting of the magnetic field in the first WP (*B*_1_) and sample distance (*L*_*s*_) to the detector are listed in the figure. (**a**) The amplitude of the polarization was obtained with sinusoidal fit and the data is corrected with *T* measured by integrating the detector and (**b**) the ratio of the visibility $(\frac{{V}_{s}}{{V}_{b}})$ was obtained by using the standard deviation of the modulation.

To verify the effectiveness of correcting the data by integrating the detector to obtain *T*, the same experiment was repeated in 2018 with a neutron monitor employed right after the sample. When the SEMSANS measurements were finished, the relative attenuation efficiency of the sample was measured again with respect to the blank using the monitor. The monitor is made of neutron active scintillator, which was designed to sample a high flux, homogenous neutron beam. As shown in Fig. 5(a) plot is corrected with *T* obtained by integrating the whole detector, the same as the previous section. Since the experiments were conducted two years later compared with Fig. 5, to our knowledge, the correlation function of the two measurements may differ from one another due to, for example, the aging or aggregation of the PMMA particles. Figure 5(b) is corrected with *T* obtained using the monitor. For both cases, the curves at different field settings overlap with each well. The difference is observed in the level of the curves at large spin echo lengths. The data corrected with the detector shows higher values, which means the measured total scattering of the sample is lower if we have to correct the data with the detector.

**Figure 6:**
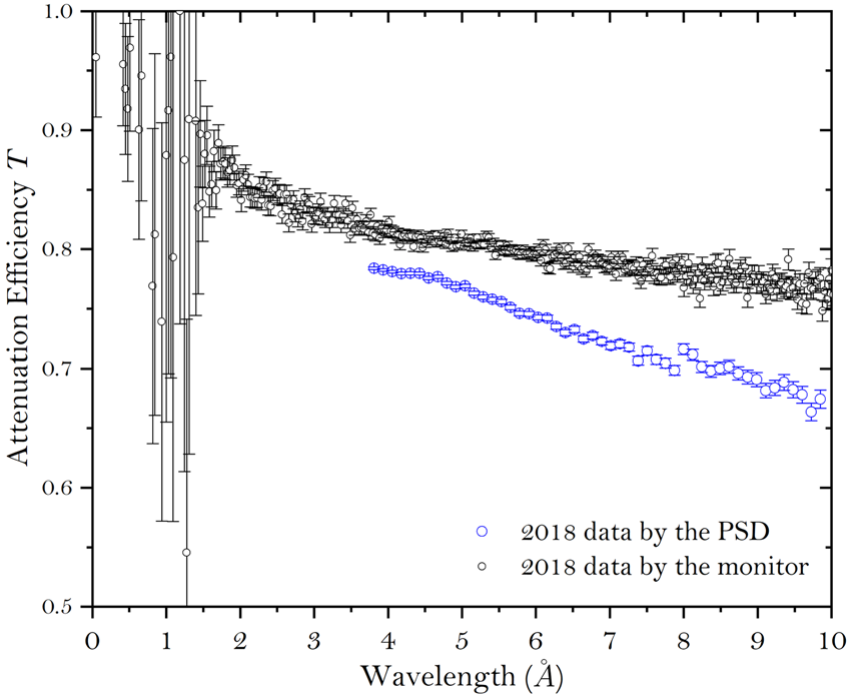
The attenuation efficiency *T* of the PMMA measured by either integrating the whole detector or the monitor.

Figure 6 shows the value of *T* measured by either integrating the whole detector or the monitor. Since the scattering angle increases as wavelength, more neutrons with long wavelength will fall off the detector of limited size than at short wavelength. Therefore the value *T* measured by the monitor is consistently higher especially for long wavelength neutrons and yields a lower value of $\frac{{\rm{\Gamma }}}{T}$, compared with that of the data measured by integrating the whole detector. This is mainly due to the difference of the solid angle coverage between the PSD and the monitor. Since the PSD is much further away, it will cover a smaller solid angle, which will miss more of the scattered neutrons especially for long wavelength neutrons. With a higher value of $\frac{{\rm{\Gamma }}}{T}$, a higher level of the curve would be expected from $\frac{\mathrm{ln}({A}_{n})}{{\lambda }^{2}\cdot t}$ as shown in Fig. 5. Since the level of the curve at long spin echo length is given by *σ* = *λ*^2^*t*Δ*ρ*^2^*ϕ*(1−*ϕ*)*ζ*, where *t* is the sample thickness, Δ*ρ* is the difference in scattering length densities between the particles and solvent *ϕ* is the volume fraction and *ζ* denotes the characteristic length scale of the sample. This means for the measurements where this level matters, using the detector with a restricted size can result in a wrong answer. While for the measurements, where only the correlation distance of the particles matters, either method can be used. To make sure one can always get the best results, a monitor is always preferred right after the sample.

## Discussion

We have discussed the feasibility of correcting the SEMSANS data to remove the contribution from the difference of the absorption efficiency between the sample and blank. The data correction is especially critical for TOF SEMSANS due to the wavelength dependence of the absorption efficiency. By employing the correction, the correlation function measured at various settings of spin echo length can overlap with each other. Though a monitor is always preferred for the measurement of the attenuation efficiency of the sample, the same high spatial resolution detector, used for the measurement of the modulation, can also be used provided its area is large enough to capture all the small angle scattered beam. But in reality, a detector with a combination of high spatial resolution and large active size is always difficult.

The correction method we have described is also applicable to any other scattering technique based on the intensity modulation, including the Talbot-Lau method and modulated intensity with zero effort (MIEZE) for quasi-elastic scattering. For the Fourier transform of both SESANS or SEMSANS, the effect of limited Q range will be presented later.

For the correction method discussed here, we are assuming the scattering is purely elastic and the neutrons will maintain their energy. But for some samples, the neutrons might gain or lose energy by inelastic scattering with the sample, which is also known as thermalization or moderation. It has been shown that, the thermalization process of cold neutrons within a liquid such as H_2_O will accelerate neutrons, causing unexpected faster neutrons^29^. The percentage of the thermalized neutrons will increase with neutron wavelength. This means that shorter wavelength neutrons may be preferable for experiments with samples that are good moderators such as H_2_O.
